# Robust Model-Based Sensor Fault Monitoring System for Nonlinear Systems in Sensor Networks

**DOI:** 10.3390/s141019138

**Published:** 2014-10-15

**Authors:** Dejun Wang, Shiyao Song

**Affiliations:** Department of Control Science and Engineering, School of Communication Engineering, Jilin University, Changchun 130025, China; E-Mail: shiyao_song1989@sina.com

**Keywords:** fault diagnosis (FD), fault regeneration, sensor network, nonlinear system, Lipschitz, equivalent model, perfect decoupling, fault monitoring system

## Abstract

A new model-based sensor fault diagnosis (FD) scheme, using an equivalent model, is developed for a kind of Multiple Inputs Multiple Outputs (MIMO) nonlinear system which fulfills the Lipschitz condition. The equivalent model, which is a bank of one-dimensional linear state equations with the bounded model uncertainty, can take the place of a plant's exact nonlinear model in the case of sensor FD. This scheme shows a new perspective whereby, by using the equivalent model, it doesn't have to study the nonlinear internal structure character or get the exact model. The influence of the model uncertainty on the residuals is explained in this paper. A method, called pretreatment, is utilized to minimize the model uncertainty. The eigenstructure assignment method with assistant state is employed to solve the problem of perfect decoupling against the model uncertainty, disturbance, system faults, the relevant actuator faults, or even the case of no input from the relevant actuator. The realization of the proposed scheme is given by an algorithm according to a single sensor FD, and verified by a simulation example. Depending on the above, a sensor fault monitoring system is established by the sensor network and diagnosis logic, then the effectiveness is testified by a simulation.

## 1. Introduction

To solve one of the critical issues surrounding the design of automatic systems, which is the systems' reliability and dependability, increasing attention should be paid to the importance of using the FD technique. It is fully integrated into vehicle control systems, robots, transport systems, power systems, process control systems, and so on [1]. In the early 1970s, the first model-based fault detection method, the so-called failure detection filter, was proposed by Beard and Jones [2]. Since then, the model-based FD theory and technique underwent rapid development.

In the early years, most model-based FD approaches were utilized in linear systems [3,4]. In the nearly two decades, lots of model-based FD methods for non-linear systems have been proposed, which can be divided into four main categories: non-linear observer-based approaches [5], filter-based approaches [6], differential geometry approaches [7] and adaptive learning approaches [8]. Although an increasing number of methods of FD for non-linear systems are addressed, the complexity and strictness of additive conditions of the existing algorithms have a strong negative influence on their applications. A FD approach which has a simple algorithm and a wide applicability for non-linear systems is required. The main reason is that the traditional methods model the whole plant and focus on the detailed internal structure, so the following problems occur:

Problem 1: it is hard to build an exact model;
Problem 2: even if it can be built, it rather difficult to design a residual generator because of the complex nonlinear structure;
Problem 3: and it is also hard to get a robust residual;
Problem 4: even if it could be designed, the algorithm might be too complex to realize in practice;
Problem 5: and it also could not handle model changes when the plant is running.

Sensor techniques attract more and more attention in automatic systems, because of their important role as the main way to get the information from the plant. The sensor FD issue plays a significant role in the FD framework and lots of research has concentrated on this field [9,10]. The current methods can be divided into two aspects. The first one is theory-based [11,12]: the mature FD theory for linear system is used to solve the MIMO nonlinear system's sensor fault and is verified by experiments. Although these methods is useful in some fields, the lack of theoretical proof of why the FD method for linear systems is suitable for nonlinear systems, makes it not rigorous, and the residual doesn't have strong robustness features. Another one is estimation-based [13]: the comparison between the measurement and its estimation according to the system dynamics is used to generate the residual. The method benefits the indication of fault or fault-free, and it depends on the data from lots of experiments, but it isn't good at presenting the fault details which is not convenient for the subsequent tasks, like fault identification, fault tolerance and fault compensation. In addition the workload is heavy.

**Figure 1.**
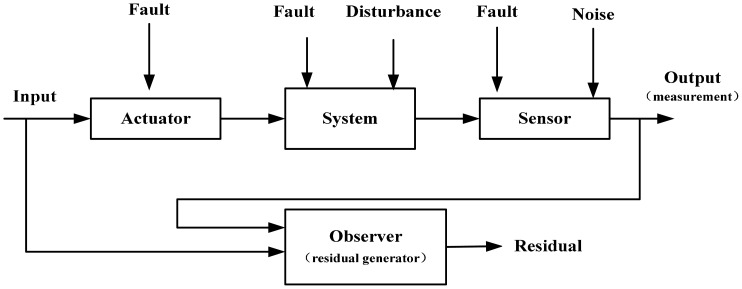
The negative influence of the residual.

In this paper, a new view for MIMO nonlinear systems fulfilling the Lipschitz condition in the circumstance of sensor fault is proposed. It is well known that the core of the model-based FD method is solving the FD problem by comparing the plant with the pre-built model. In linear system FD problems, it is clearly claimed in Ding's paper [14] that the function of the observer in a model-based method is only as an output observer. According to this important theory, the new view not only combines the advantages of the above two methods in the sensor FD field, but also solves the above five problems in nonlinear system FD issues:

(1) For problem 1: to solve a single sensor fault, an equivalent model is used to take the place of the exact model of the plant.
  The models used in FD are usually set up according to physical or mathematical theories. The process needs to know many details about the dynamics and their relationships. This style of model would be called dynamics-based model (DBM). The core of DBM is starting the FD from the system's internal structure. It could describe the dynamics of the system properly, but usually it isn't suitable for FD, especially for non-linear systems. In addition it often can't solve the robustness problem of the residual, even to design a residual generator. Currently, some researches focus on dealing with only several typical parts in one paper, like the Itô stochastic systems [15], time-delay system [16], T-S fuzzy system [17] and so on. For a general nonlinear system, in which we cannot detect the exact sort of internal structure, these methods fail. In fact, the real non-linear plant is too hard to model. Hence, building a model which is suitable for FD is necessary. It can be called fault-based model (FBM).
  In this paper, aiming at the sensor FD, a FBM is established, which uses the measurements from the sensors in the plant as the states and the output is the sensors' measurements. Depending on one FBM, an equivalent model is established only for a single sensor fault. The equivalent model is transformed identically from the common style of nonlinear system fulfilling the Lipschitz condition, so, to some extent, the equivalent model is an exact model. Here, we don't have to study the nonlinear internal structure character or get the exact model of nonlinear systems. That means, the equivalent model not only is a exact model for sensor FD issue, but also will benefit the following FD process a lot.
(2) For problem 2: because of the clear and simple structure of the equivalent model, the mature FD theory for linear system could be used directly.
  For the DBM method, it must give the special feedback facing the typical parts. Like the sliding mode approach is used to deal with a class of Itô stochastic systems, the descriptor observer design method is used to handle time-delays. Although these methods are useful, the point of focusing on typical parts makes it quite difficult to design a widely-used method for general nonlinear systems.
  The equivalent model has a clear and simple structure-for-single-sensor which is just a one-dimension linear state equation with the model uncertainty. Although the details about the model uncertainty are unknown, we can confirm that they are combined in the certain position in model the uncertainty and the model uncertainty is bounded. Thus, depending on considering the model uncertainties as disturbances [18,19], lots of FD methods for linear system could be used directly and easily. That means, it is quite easy to design an output observer for the equivalent model to generate residual, so the common method for linear system (this paper uses the linear Luenberger observer) could be used widely for general nonlinear systems.
(3) For problem 3: use the perfect decoupling method and get the strong robust residual
  A model-based FD for actual plants strongly needs a robust residual generator. A robust residual generator means that the obtained residual should be sensitive to the faults and insensitive to the disturbances, model uncertainty and any other negative influence (shown in Figure 1). The robust control-based theory in handling the model uncertainties plays a significant role in this field, like the LMI approach [20], and norm-based method [21], but they could only, to some extent, reduce the negative influence, and can't make the perfect decoupling come true. The famous approaches are unknown input observer [22] and eigenstructure assignment [23–25] which will realize it very well. In this paper, the eigenstructure assignment method is utilized to solve the problem and the assistant state is introduced to give a hand for realization. On the other hand, the proposed method could ignore the negative influence caused by the relevant actuator fault or even no input from the relevant actuator. Subsequently, the target sensor's fault could be detected successfully in theory.
  In practice, the electronic control unit can only solve a discrete signal. The eigenstructure assignment method realizes the perfect decoupling by making the transfer function from disturbance to residual equal to 0. Hence, there is a potential risk that the transfer function is not equal to 0 when the transfer occurs between continuous signals and digital signals. The model uncertainty's influence may be unbounded regarding residuals generated with output observer, which is determined by the parameters of output observer. A method, called pretreatment, that involves choosing the proper parameters of the diagnosis observer to minimize the model uncertainty is raised in this paper. It could prevent the negative influence on the robust performance in the residual because of the error caused by signal transfers or model uncertainty.
(4) For problem 4: the simple structure of observer
  For the typical parts discussed in the above, the proposed methods, like sliding mode approach, the descriptor observer and so on, have complex structures and lots of parameters need to be designed. The analysis redundant component in this paper is only a 2-dimensional linear observer for a single sensor fault, which has four parameters to be designed. The gain of the observer could be small enough to be realized in an electronic control unit. Therefore, comparing the current four main sorts methods presented in the above, the method in this paper benefits the practice very much.
(5) For problem 5: the basis of the equivalent model
  The exact model must be changed when it is running, like the time-delay part's parameters may change, T-S fuzzy system could be more complex and could described as before. This time, the former useful methods fail. The reason is this category depends on the exact internal structure of plant.
  The equivalent model is transformed identically from the common style of nonlinear system fulfilling the Lipschitz condition. It's a fuzzy description of the system but has a clear framework.
  When the exact model has changed, the framework won't change. The only impact on equivalent model is the model uncertainty, which could be handled by pretreatment and perfect decoupling. The management concentrates on the feature of the system's output (in order to design an output observer), instead of the internal structure. Thus, this method could handle the change of model when the plant is working on-line.
  The above discussion could handle the single sensor fault successfully. With this method and the view of multi-sensor fusion, a plant's sensor fault monitoring system could be set up for all sensors' fault diagnosis and even for fault-tolerance and each sensor's fault could be isolated easily and natively.
  The paper is organized as follows: in Section 2, the process of building the equivalent model is proposed. In Section 3, the sensor FD problem is formulated, and the influence of the model uncertainty is explained. A method, called pretreatment, that involves choosing the proper parameters of the diagnosis observer to minimize the model uncertainty, is shown. Section 4 provides the perfect decoupling against the disturbance, the model uncertainty and the relating actuator with the eigenstructue assignment method and the help of the assistant state. Section 5 provides one algorithm for single sensor FD and a simulation example. In Section 6, a sensor fault monitoring system is established for all sensors' diagnosed faults and fault tolerance and the effectiveness is showed by a simulation.

## 2. Equivalent Model

### 2.1. The Establishment of FBM

The general model-based FD needs the system's exact model and this is very difficult to get. The process of modeling is generally based on physics or mathematics, but the model could hardly describe the system accurately. For instance, a vehicle model is built, which depends on the vehicle lateral dynamics and Laplace Transformation, to diagnosis the yaw rate sensor fault. The model couldn't express all details of the vehicle, even only the lateral dynamic. Generally speaking, the DBM could be written as follow (showing in Figure 1):

(1) $$\{\begin{array}{c} \dot{x}(t)=\bar{A}x(t)+\bar{B}u(t)+E_{\tilde{d}}\tilde{d}+E_{f_{a}}f_{a}+E_{f_{s}}f_{s}\\ y(t)=\bar{C}x(t)+\bar{D}u(t)+F_{\tilde{d}}\tilde{d}+F_{f_{a}}f_{a}+F_{f_{s}}f_{s} \end{array}$$

where *Ā* = *A* + Δ*A* + Δ*A_f_*, *B̄* = *B* + Δ*B* + Δ*B_f_*, *C̄* = *C* + Δ*C* + Δ*C_f_, D̄* = *D*+Δ*D*+Δ*D_f_. x*(*t*) ∈ ℜ*^n^* denotes the state vector, *u*(*t*) ∈ ℜ*^m^* is the input vector, *y*(*t*) ∈ ℜ*^n^* is the output vector. *A, B, C* and *D* are the linear system matrices with proper dimensions. Δ*A*, Δ*B*, Δ*C* and Δ*D* are model uncertainty, Δ*A_f_*, Δ*B_f_*, Δ*C_f_* and Δ*D_f_* are system faults, *d̃*, *f_a_* and *f_s_* are disturbance, actuator fault and sensor fault, *E_d̃_*, *E_fa_, E_fs_, F_d_, F_fa_* and *F_fs_* are their distribution matrixes with proper dimensions, respectively.

The DBM is a proper expression of the system's dynamic characters and structure features, but usually isn't suitable for FD, especially for the non-linear system. Because of the complex details of DBM, it's rather hard to generate a robust residual or even design a residual generator, so the FBM strongly aiming at FD is needed. It's an exact model of system and could be known as a preparation of FD. In this paper, to diagnose the sensor's fault, a method, setting up a FBM, which is that the dynamics measuring by all sensors, needing to be diagnosed, are used as state and the outputs are the measurements of all sensors, could be described as follows:

(2) $$\{\begin{array}{c} \dot{x}(t)=\bar{F}x(t)+\bar{G}u(t)+E_{\tilde{d}}\tilde{d}+E_{f_{a}}f_{a}\\ y(t)=x(t)+f_{s} \end{array}$$

where *F̄* = *F* + Δ*F* + Δ*F_f_, Ḡ* = *G* + Δ*G* + Δ*G_f_, F* and *G* are the linear system matrices with proper dimensions. Δ*F* and Δ*G* denote the model uncertainties, Δ*F_f_* and Δ*G_f_* describe the system faults. The essential of this method is the optimal sensor configuration, which is used to realize that selection of a minimum number of sensors to obtain the maximum amount of information for reliable state estimation in automotive applications in modeling. In this paper, it is used to benefit the sensor FD: although (1) and (2) are both exact models of the system, the results of the comparison between (1) and (2) could be showed straightforwardly that, in (2), the sensor faults have a conspicuous position which make it much easier to design a robust residual generator than in (1).

### 2.2. Describe the Target State by a Simple Equation

The general model-based FD scheme would design a residual generator on (2) straightforwardly. This time, the problem of fault isolation is unavoidable and it will also exert a negative influence on fault identification, especially for the case that the number of sensors is large. Hence, the FD approach to set up a residual generator only for one sensor that is called target sensor at one time, will be effective. The FD methods for non-linear system are developing rapidly, but are not convenient to be utilized directly, whereas the methods for linear systems are well-established. It will be very convenient and effective using the FD method of linear systems to solve the problem of non-linear systems, if the nonlinear system could be transformed into the form of a linear system with bounded model uncertainty. The model uncertainty could be decoupled from the residual as a disturbance. This is actualized in this paper.

To facilitate the realization of the above, the preparation is addressed in the section. Neglecting the faults and disturbance, (2) could be transformed into:

(3) $$\{\begin{array}{l} \dot{x}(t)=\bar{\bar{F}}x(t)+\bar{\bar{G}}u(t)\\ y(t)=x(t) \end{array}$$

where *F̿=F* + Δ*F* , *G̿*=*G*+Δ*G*, and it fulfills the Lipschitz condition.

The exact model of (3) can be described by:

(4) $$\{\begin{array}{lll} \left[\begin{array}{c} {\dot{x}}_{1}(t)\\ {\dot{x}}_{2}(t)\\ \vdots \\ {\dot{x}}_{n}(t) \end{array}\right] & = & \left[\begin{array}{cccc} f_{11} & f_{12} & \cdots & f_{1n}\\ f_{21} & f_{22} & \cdots & f_{2n}\\ \vdots & \vdots & \ddots & \vdots \\ f_{n1} & f_{n2} & \cdots & f_{nn} \end{array}\right]\left[\begin{array}{c} x_{1}(t)\\ x_{2}(t)\\ \vdots \\ x_{n}(t) \end{array}\right]+\left[\begin{array}{cccc} g_{11} & g_{12} & \cdots & g_{1m}\\ g_{21} & g_{22} & \cdots & g_{2m}\\ \vdots & \vdots & \ddots & \vdots \\ g_{n1} & g_{n2} & \cdots & g_{nm} \end{array}\right]\left[\begin{array}{c} u_{1}(t)\\ u_{2}(t)\\ \vdots \\ u_{m}(t) \end{array}\right]\\ \left[\begin{array}{c} y_{1}(t)\\ y_{2}(t)\\ \vdots \\ y_{n}(t) \end{array}\right] & = & \left[\begin{array}{cccc} 1 & 0 & \cdots & 0\\ 0 & 1 & \cdots & 0\\ \vdots & \vdots & \ddots & \vdots \\ 0 & 0 & \cdots & 1 \end{array}\right]\left[\begin{array}{c} x_{1}(t)\\ x_{2}(t)\\ \vdots \\ x_{n}(t) \end{array}\right] \end{array}$$

where *x_i_*(*t*) is the *i*th state of *x*(*t*), *ul*(*t*) is the *l*th input of *u*(*t*), *y_i_*(*t*) is the *i*th output of *y*(*t*), *i*, *j*, *h*∈[0,*n*],*l*∈[0,*m*] , *f_ij_* and *g_hl_* are the function of *x*_1_, *x_2_*,…,*x_n_* and *t* respectively, namely, *f_ij_*: *f_ij_* (*x*_1_, *x*_2_, … *x_n_*, *t*) and *g_hl_:g_hl_* (*x*_1_*,x_2_*,…*x_n_,t*). Since *x_i_*(*t*) is the function of *t*, it means *f_ij_*:*f_ij_*(*t*) and *g_hl_* : *g_hl_*(*t*) Hence, we could describe everyone of *f_ij_* and *g_hl_* by an expression including *t* and a constant. In another word, (2) could be also described as follows:

(5) $$\{\begin{array}{lll} \left[\begin{array}{c} \overset{\cdot }{x_{1}}(t)\\ \overset{\cdot }{x_{2}}(t)\\ \vdots \\ \overset{\cdot }{x_{n}}(t) \end{array}\right] & = & \left[\begin{array}{cccc} a_{11}+\Delta a_{11} & a_{12}+\Delta a_{12} & \cdots & a_{1n}+\Delta a_{1n}\\ a_{21}+\Delta a_{21} & a_{22}+\Delta a_{22} & \cdots & a_{2n}+\Delta a_{2n}\\ \vdots & \vdots & \ddots & \vdots \\ a_{n1}+\Delta a_{n1} & a_{n2}+\Delta a_{n2} & \cdots & a_{nn}+\Delta a_{nn} \end{array}\right]\left[\begin{array}{c} x_{1}(t)\\ x_{2}(t)\\ \vdots \\ x_{n}(t) \end{array}\right]+\left[\begin{array}{cccc} b_{11}+\Delta b_{11} & b_{12}+\Delta b_{12} & \cdots & b_{1m}+\Delta b_{1m}\\ b_{21}+\Delta b_{21} & b_{22}+\Delta b_{22} & \cdots & b_{2m}+\Delta b_{2m}\\ \vdots & \vdots & \ddots & \vdots \\ b_{n1}+\Delta b_{n1} & b_{n2}+\Delta b_{n2} & \cdots & b_{nm}+\Delta b_{nm} \end{array}\right]\left[\begin{array}{c} u_{1}(t)\\ u_{2}(t)\\ \vdots \\ u_{m}(t) \end{array}\right]\\ \left[\begin{array}{c} y_{1}(t)\\ y_{2}(t)\\ \vdots \\ y_{n}(t) \end{array}\right] & = & \left[\begin{array}{cccc} 1 & 0 & \cdots & 0\\ 0 & 1 & \cdots & 0\\ \vdots & \vdots & \ddots & \vdots \\ 0 & 0 & \cdots & 1 \end{array}\right]\left[\begin{array}{c} x_{1}(t)\\ x_{2}(t)\\ \vdots \\ x_{n}(t) \end{array}\right] \end{array}$$

where *a_ij_* and *b_hl_* are suitable constants, *i, j, h* ∈ [0,*n*] , *l* ∈ [0,m] . Δ*a_ij_*:Δ*a_ij_*(*x*_1_, *x*_2_,*…x_n_*,*t*) , Δ*b_hl_*:Δ*b_hl_* (*x*_1_*, x*_2_,…*x_n_,t*). And it also means Δ*a_ij_*:Δ*a_ij_*(*t*) and Δ*b_hl_* : Δ*b_hl_* (*t*). They fulfill the relationships which are Δ*a_ij_* = *f_ij_* − *a_ij_* and Δ*b_hl_* = *g_hl_* − *b_hl_*, respectively.

The models (4) and (5) are both the descriptions of the MIMO non-linear system (3). The difference between them is the style of description. (4) uses *f_ij_* and *g_hl_*, but (5) uses *a_ij_* , *b_hl_,* Δ*a_ij_* and Δ*b_hl_*. Δ*a_ij_* and Δ*b_hl_* are model uncertainties. After the model uncertainties are taken out, (5) becomes a linear system as follows:

(6) $$\{\begin{array}{lll} \left[\begin{array}{c} {\dot{x}}_{1}(t)\\ {\dot{x}}_{2}(t)\\ \vdots \\ {\dot{x}}_{n}(t) \end{array}\right] & = & \left[\begin{array}{cccc} a_{11} & a_{12} & \cdots & a_{1n}\\ a_{21} & a_{22} & \cdots & a_{2n}\\ \vdots & \vdots & \ddots & \vdots \\ a_{n1} & a_{n2} & \cdots & a_{nn} \end{array}\right]\left[\begin{array}{c} x_{1}(t)\\ x_{2}(t)\\ \vdots \\ x_{n}(t) \end{array}\right]+\left[\begin{array}{cccc} b_{11} & b_{12} & \cdots & b_{1m}\\ b_{21} & b_{22} & \cdots & b_{2m}\\ \vdots & \vdots & \ddots & \vdots \\ b_{n1} & b_{n2} & \cdots & b_{nm} \end{array}\right]\left[\begin{array}{c} u_{1}(t)\\ u_{2}(t)\\ \vdots \\ u_{m}(t) \end{array}\right]\\ \left[\begin{array}{c} y_{1}(t)\\ y_{2}(t)\\ \vdots \\ y_{n}(t) \end{array}\right] & = & \left[\begin{array}{cccc} 1 & 0 & \cdots & 0\\ 0 & 1 & \cdots & 0\\ \vdots & \vdots & \ddots & \vdots \\ 0 & 0 & \cdots & 1 \end{array}\right]\left[\begin{array}{c} x_{1}(t)\\ x_{2}(t)\\ \vdots \\ x_{n}(t) \end{array}\right] \end{array}$$

In (6), to some extent, *a_ij_* and *b_hl_* become the description of linear relationship of (4), so (6) is a linear system that couldn't describe (3) exactly, but (4) could. The Δ*a_ij_* and Δ*b_hl_* represent the model uncertainty between (4) and (6).

Remark 1: In the condition of choosing the same states, for the same nonlinear plants, the linear model couldn't provide a complete description like the exact nonlinear model. The model uncertainty exists.

Theory 1: By choosing one state from (3) arbitrarily, the corresponding exact model in (3) could be written by a one-dimension state equation including model uncertainty (like (7) or (8)), with one or some inputs from (3). The model uncertainty is a unknown function of *t*, which is an implicit description that maybe cannot be described by one certain expression:

(7) $$\{\begin{array}{l} \overset{\cdot }{x_{1}}(t)=ax_{i}(t)+\vec{b_{v}}\vec{u_{v}}+M_{i}\\ y(t)=x_{i}(t) \end{array}$$

(8) $$\{\begin{array}{l} \overset{\cdot }{x_{i}}(t)=ax_{i}(t)+bu_{l}(t)+M_{i}\\ y(t)=x_{i}(t) \end{array}$$

where *x_i_* denotes any one state of (3), *a*, *b* and *v* are constants and *a* ≠ 0 , *v* ∈ [1, *m*] , *b⃗_v_* is a *v*-dimensions row vector, *u⃗_v_* is a v-dimensions column vector existing in *u*(*t*). *M_i_* is the model uncertainty and is bounded.

Proof: Towards *x_i_*, any one state of (5), the whole mathematical description is:

$$\begin{array}{l} \overset{\cdot }{x_{i}}=\sum_{j=1}^{n}\left(a_{ij}+\Delta a_{ij}\right)x_{j}+\sum_{l=1}^{n}\left(b_{il}+\Delta b_{il}\right)u_{l}\\ =a_{ii}x_{i}+\vec{b_{v}}\vec{u_{v}}+(\Delta a_{ii}x_{i}+\Delta \vec{b_{v}}\vec{u_{v}})+[\sum_{j=1,j\neq i}^{n}(a_{ij}+\Delta a_{ij})x_{j}+(b_{\left(m-v\right)}^{\to }n+\Delta b_{\left(m-v\right)}^{\to })u_{\left(m-v\right)}^{\to }] \end{array}$$

where *b⃗_i_* ∈ *ℜ^m^*, Δ*b⃗_i_* ∈ *ℜ^m^*, *b⃗_v_* ∈ *ℜ^v^*, Δ*b⃗_v_* ∈ *ℜ^v^*, $b_{\left(m-v\right)}^{\to }$ ∈ *ℜ^m−v^*, Δ$b_{\left(m-v\right)}^{\to }$ ∈ *ℜ^m−v^* are row vectors, *u⃗_v_* ∈ *ℜ^v^*, $u_{\left(m-v\right)}^{\to }$ ∈ *ℜ^m−v^* are column vectors, *b⃗_i_* = [*b*_*i*1_ *b*_*i*2_ … *b_im_*], Δ*b⃗_i_* = [Δ*b*_*i*1_ Δ*b*_*i*2_ … Δ*b_im_*]. *u⃗_v_* and $u_{\left(m-v\right)}^{\to }$ consist of arbitrary *v* inputs and the remaining inputs from *u*(*t*), respectively. *b⃗_v_* and Δ*b⃗_v_* consist of the elements, the corresponding elements in *u⃗_v_*, from *b⃗_i_* and Δ*b⃗_i_*, respectively. $b_{\left(m-v\right)}^{\to }$ and Δ$b_{\left(m-v\right)}^{\to }$ consist of the elements, the corresponding elements in $u_{\left(m-v\right)}^{\to }$, from *b⃗_i_* and Δ*b⃗_i_*, respectively.

By setting:

$$\begin{array}{c} M_{i1}=\Delta a_{ii}x_{i}+\Delta \vec{b_{v}}\vec{u_{v}}\\ M_{i2}=\sum_{j=1,j\neq i}^{n}(a_{ij}+\Delta a_{ij})x_{j}+(b_{\left(m-v\right)}^{\to }+\Delta b_{\left(m-v\right)}^{\to })u_{\left(m-v\right)}^{\to }\\ M_{i}=M_{i1}+M_{i2} \end{array}$$

where *M_i_* denotes the model uncertainty. The model uncertainty *M_i_* is made up with *M_i_*_1_ which denotes the incomplete description by linear part, and *M_i_*_2_ which denotes the influence from state and input that not be modeled.

Because of the Lipschitz condition, there exists a constant Lipschitz *γ*, fulfills:

(9) $$\left\|\Phi (x_{1},u)-\Phi (x_{2},u)\right\|\leq \gamma \left\|x_{1}-x_{2}\right\|$$

where Φ(*x, u*)=Δ*Ax* + Δ*Bu*.

So, there exists a set ℂ, which ℂ = {*x*| *x* ∈ ℂ} and *x* is continuous differentiability, fulfills (9) and

$$\underset{x_{1}\to x_{2}}{\lim}(\Phi (x_{1},u)-\Phi (x_{2},u))=0$$

where *u* ≤ *u*_max_ , *M_i_* ≤ *M_i__*_max_. *umax* and *Mi*_max are constants. So, *Mi* is bounded.

Subsequently, (3) could be transformed into:

(10) $$\{\begin{array}{l} \overset{\cdot }{x_{i}}(t)=a_{ii}x_{i}(t)+\vec{b_{v}}\vec{u_{v}}+M_{i}\\ y(t)=x_{i}(t) \end{array}$$

when *v* = 1 (we choose only one input):

(11) $$\{\begin{array}{l} \overset{\cdot }{x_{i}}(t)=a_{ii}x_{i}(t)+b_{il}u_{l}(t)+M_{i}\\ y(t)=x_{i}(t) \end{array}$$

### 2.3. Establishment of the Equivalent Model

Through the above Theory (1), a single sensor FD concerning one state *x_i_* of a non-linear system in (3), could be regarded as the state *x_i_* of a one-dimension linear system in (8). Based on this, the equivalent model could be established which is identical transformation of (3) and is also an exact model.

Set up of a fault model matching (8) is:

(12) $$\{\begin{array}{l} \overset{\cdot }{x_{i}}(t)=(a+\Delta a_{f})x_{i}(t)+(b+\Delta b_{f})(u_{l}(t)+f_{a})+M_{i}+\tilde{d}\\ y(t)=x_{i}(t)+f_{s} \end{array}$$

where Δ*a_f_* and Δ*b_f_* denote system faults, they are bounded normally.

By setting*d* = Δ*a_f_x_i_*(*t*) + Δ*b_f_*(*u_l_*(*t*) + *f_a_*)*M_i_* + *d̃* + *bf_a_,* (12) is written by:

(13) $$\{\begin{array}{l} \overset{\cdot }{x_{i}}(t)=ax_{i}(t)+bu_{l}(t)+d\\ y(t)=x_{i}(t)+f_{s} \end{array}$$

Remark 2: *d* is called the equivalent disturbance. It is a combination including the factors which exert negative influences on FD, and it is reasonable to assume that *d* is bounded normally.

Remark 3: In the problem of sensor FD of *x_i_*, (13) is called the equivalent model of (2), which is used to take the place of (2) to work. Hence, facing the problem of sensor FD of a nonlinear system, the equivalent model (13) is easily used instead of considering the internal structure's character into (2).

Remark 4: In the above analysis, a new view could be adopted, that setting up one equivalent model instead of the original plant, participates in the process of FD. The equivalent model could be a low level linear system with model uncertainty. Although we don't know whether the model uncertainty could be addressed by one explicit expression, the equivalent model is exact enough in the case of FD. Hence, we can understand it as that the equivalent model is exact but have an implicit part in it.

Remark 5: Among the approaches of model-based FD, the most important issue is that it hardly gets the exact model of the plant. The way of building an equivalent model, to some extent, solves the problem.

## 3. Problem Formulation

### 3.1. The Observer-Based Approach

The basic work of model-based FD is the design of residual generator which is a redundancy component. For our purpose of residual generation, known as a comparison between system measurements and their redundancy, the residual generator is understood as a reconstruction of the measured quantities of the system under consideration. The main methods are fault detection filter (FDF), diagnostic observer (DO) and parity space. Here, the classical observer-based approach is used to detect the sensor fault. The observer used in FDF is seen as a state observer, but in this paper, it only acts as an output observer. The observer for (13) is:

(14) $$\{\begin{array}{l} {\dot{\hat{x}}}_{i}(t)=a{\hat{x}}_{i}(t)+bu_{l}(t)+k(y(t)-\hat{y}(t))\\ \hat{y}(t)={\hat{x}}_{i}(t) \end{array}$$

The residual is:

(15) $$r(s)=w(y(s)-\hat{y}(s))=G_{rd}(\text{s})d+G_{rf_{s}}(s)f_{s}$$

where *x̂_i_*(*t*) and *ŷ* (*t*) are the estimations of *x_i_* an*d y*(*t*), *k* is the observer gain to be designed, *w* is the weighting gain of the residual, *G_rd_* (*s*) and *G_rfs_*(*s*) are transfer functions of *d* and *f_s_* to *r*, respectively.

Theory 2 [2]: Given a linear system (16) as follow:

(16) $$\{\begin{array}{l} \overset{\cdot }{x(t)}=Ax(t)+Bu(t)+E_{d}d+E_{f}f\\ y(t)=Cx(t)+Du(t)+F_{d}d+F_{f}f \end{array}$$

where *x*(*t*) ∈ ℜ*^n^*, *y*(*t*) ∈ℜ*^n^*, *d* is the unknown input, *f* is the fault, *E_d_*, *E_f_*, *F_d_* and *F_f_* are their distribution matrices. Then the perfect unknown input decoupling condition is, if and only if:

$$rank\left[\begin{array}{ll} A-sI & E_{d}\\ C & F_{d} \end{array}\right]<rank\left[\begin{array}{lll} A-sI & E_{f} & E_{d}\\ C & F_{f} & F_{d} \end{array}\right]\leq n+m$$

Proof: see reference [2].

Remark 6: according to Theory (2), in the residual *r* generated from (15) with (13) and (14), it is impossible for the perfect decoupling of the disturbance *d,* no matter what *k* and *w* are. How to get the perfect decoupling will be shown in Section 4.

### 3.2. About the Model Uncertainty

The robust FD requires that the residual is sensitive to the fault occurrence and insensitive to the disturbance, model uncertainty and any other negative influences. Generally speaking, there are two ways to approach this problem proposed in [26]: increasing the robustness of the detection system by using advanced robust FDI theory, and making use of additional information (extending the model or establishing adaptive thresholds). Considering the model uncertainty shown in (13), we know nothing but a function of *t* normally. It means it fails to use the second method and design a perfect decoupling method is necessary. Thus, the first approach is adopted and will be addressed in the Section 4. On the other hand, facing with the problem of realization in electronic control unit, we would do some pretreatment about choosing the parameters of the observer to reduce (minimize) the influence of model uncertainty on residual. It will be explained in the next section.

### 3.3. The Approach of Minimizing Model Uncertainty's Influence

Through minimizing the influence of model uncertainty on residual, *M_i_* is tried to be small as possible in residual.

Theory 3: By bringing the proper compensations in the parameters *a* and *b* of the observer (14), the influence of model uncertainty *M_i_* on residual could be minimized.

Proof: Put the compensations Δ*a* and Δ*b* in (14):

(17) $$\{\begin{array}{l} {\dot{\hat{x}}}_{i}=(a+\Delta a){\hat{x}}_{i}+(b+\Delta b)u_{l}+k(y-\hat{y})\\ \hat{y}={\hat{x}}_{i} \end{array}\Rightarrow \{\begin{array}{l} \dot{\dot{\hat{x}}}=\bar{a}{\hat{x}}_{i}+\bar{b}u_{l}+k(y-\hat{y})\\ \hat{y}={\hat{x}}_{i} \end{array}$$

where Δ*a* and Δ*b* satisfy that *ā* = *a* + Δ*a*, *b̄* = *b* + Δ*b* .

And make an equivalent transformation from (8) as follows:

(18) $$\{\begin{array}{l} {\dot{x}}_{i}=(a+\Delta a)x_{i}+(b+\Delta b)u_{l}+[M_{i}-(\Delta ax_{i}+\Delta bu_{l})]\\ y=x_{i} \end{array}=\{\begin{array}{l} {\dot{x}}_{i}=\bar{a}x_{i}+\bar{b}u_{l}+[M_{i}-(\Delta ax_{i}+\Delta bu_{l})]\\ y=x_{i} \end{array}$$

Then, using (17) as an observer to observe (18), the influence of model uncertainty on the residual becomes:

(19) $${M^{\prime }}_{i}=M_{i}-(\Delta ax_{i}+\Delta bu_{l})$$

So, by choosing the proper compensations Δ*a* and Δ*b,* $M_{i}^{\prime }$ will be minimized when Δ*ax_i_* + Δ*bu_v_* as close as possible towards *M_i_*.

Remark 7: $M_{i}^{\prime }$ denotes the influence from *M_i_* on residual. According to Theory (3), there always exists the proper Δ*a* and Δ*b* to minimize the $M_{i}^{\prime }$ , whatever *a* and *b* are. Hence, we can ignore the influence of the internal structure character in (3) at the time of designing the parameters of (14), and the only thing is how to find *ā* and *b̄*.

Remark 8: The model uncertainty *M_i_* is added to the observer (14). By the proper compensations in the parameters of the observer (14), the observational ability of observing the internal details in (8) will be enhanced, and the negative influence of the model uncertainty will be mitigated. It also occurs that the size of $M_{i}^{\prime }$ will be enormous or unbounded by unsuitable *ā* and *b̄*, which is the first main reason to do some pretreatment proposed in 3.2.

Remark 9: The important conclusion, which designing the proper parameters of (17) could ignore the internal structure characteristic of (2), will bring a profound convenience when we face the large complex plant.

The work of designing the parameters of (17) should be done before the design of residual generator of FD. Here, it is called pretreatment.

Theory 4: Making *y* − *ŷ* as small as possible, means making $M_{i}^{\prime }$ as small as possible.

Proof: Assume that we have already finished the pretreatment, and the model and the observer are (18) and (17), the model uncertainty is $M_{i}^{\prime }$

It exists:

(20) $$\frac{d}{dt}x_{i}-\frac{d}{dt}{\hat{x}}_{i}=(\bar{a}-k)(x_{i}-{\hat{x}}_{i})+{M^{\prime }}_{i}$$

Let *e_y_* = *y* − *ŷ* = *x*_i_ − *x̂_i_*, *ā* − *k* is stable, then:

(21) $${M^{\prime }}_{i}=\frac{d}{dt}e_{y}-(\bar{a}-k)e_{y}$$

So:

(22) $$e_{y}=y-\hat{y}=e^{(\bar{a}-k)t}e^{y}(t_{0})+\int_{t_{0}}^{t}e^{\left(\bar{a}-k)(t-\tau \right)}{M^{\prime }}_{i}d\tau$$

where, *t0* is the initial time. To make $M_{i}^{\prime }$ as small as possible means to make *e_y_* = *y* − *ŷ* as small as possible through choosing the proper Δ*a* and Δ*b*.

From Theory (4), an algorithm, called table approach, could be used to minimize the influence of model uncertainty on the residual. It is an approach that chooses the relating parameters from the pre-structured table including all pre-calculated and relating parameters that the system needs. It is widely used in the engineering practice. The procedure in detail is done as follows:

Algorithm 1:Step 1: set a table including all the pre-calculated and relating parametersStep 2: choose the parameters when *e_y_* meets theory 4.Step 3: stop


## 4. Solution

### 4.1. Eigenstructure Assignment Approach

The robustness issues of the residual have attracted much attention following the study on the design of residual generators. Generally, the generator can't avoid the influence of the disturbance. Subsequently, it should be satisfied that the residual is sensitive to the fault and insensitive to the disturbance, model uncertainty and any other negative influence. A number of methods have been proposed, like the LMI approach and norm-based method. Although the negative influence is reduced, in addtion, if the generated residual is independent not only of the inputs and initial conditions but also the unknown input, then it can be directly used as a fault indicator and the robustness issues will be solved completely. This concept is called perfect decoupling. The famous approaches are eigenstructure assignment and unknown input observer. In the paper, the eigenstructure assignment method is utilized to actualize the perfect decoupling.

Consider a linear system including sensor fault and disturbance, which is described by:

(23) $$\{\begin{array}{l} \overset{\cdot }{x}=Ax+Bu+Ed\\ y=Cx+fs \end{array}$$

where *f_s_* is the sensor fault, *d* is the disturbance and its distribution matrix *E* is assumed to be known. *A, B* and *C* are known system matrices with proper dimensions.

Set up a linear observer in the form of:

(24) $$\{\begin{array}{l} \dot{\hat{x}}=A\hat{x}+Bu+K(y-\hat{y})\\ \hat{y}=C\hat{x} \end{array}$$

where *K* is the observer gain, and it should make *A_c_* = *A* − *KC* stable.

So, the residual is:

(25) $$r(s)=W(y-\hat{y})=G_{rd}(s)d+G_{rf_{s}}f_{s}$$

where *W is* a weighting matrix, *G_rd_*(*s*) = *WC*(*sI* − *A_c_*)^−1^*E*, *G_rfs_* = −*WC*(*sI*−*A_c_*)^−1^*K* + *W.* The disturbance decoupling condition is *Grd*(*s*) = 0.

The problem of robust FD becomes to find *W* and *K* such that *G_rd_*(*s*) = 0 is satisfied and *Ac* is stable, and the following result exists.

Lemma 1: If *WCE* = 0 and all rows of the matrix *WC* are left eigenvectors of *Ac* corresponding *to p* eigenvalues of *Ac*, then *G_rd_*(*s*) = 0. *p* is the dimension of the residual.

Proof: see [2].

Now, the perfect decoupling method has been introduced in 3.2 and the problem of realization in practice occurs. The electronic control unit (ECU) could only deal with discrete signals. Hence, there is a potential risk that the transfer function is not equal to 0 when the transform is done between continuous signal and digital signals. Although it may be small, it will have negative influence on the robust performance when the size of $M_{i}^{\prime }$ is big enough, which is the second main reason to do some pretreatment proposed in Section 3.2.

### 4.2. The Sensor FD Based on Assistant States

In order to implement the sensor FD of state *x_i_*, *x̃_i_* is needed to be defined as the assistant state. Assume it could be measured by a sensor and ${\dot{\tilde{x}}}_{i}=x_{i}$ . Combining ${\dot{\tilde{x}}}_{i}=x_{i}$ and (13), we get:

(26) $$\{\begin{array}{l} \left[\begin{array}{c} {\dot{\tilde{x}}}_{i}\\ \cdot \\ x_{i} \end{array}\right]=\left[\begin{array}{ll} 0 & 1\\ 0 & a \end{array}\right]\left[\begin{array}{c} {\tilde{x}}_{i}\\ x_{i} \end{array}\right]+\left[\begin{array}{c} 0\\ b \end{array}\right]u_{l}+\left[\begin{array}{c} 0\\ 1 \end{array}\right]d\\ \left[\begin{array}{c} y_{1}\\ y_{2} \end{array}\right]+\left[\begin{array}{c} {\tilde{x}}_{i}\\ x_{i} \end{array}\right]+\left[\begin{array}{c} {\tilde{f}}_{s}\\ f_{s} \end{array}\right] \end{array}$$

where *f̃*_s_ denotes the sensor fault of *x̃_i_*.

Then (26) can be rewritten by:

(27) $$\{\begin{array}{l} \overset{\cdot }{x}=Ax+Bu_{l}+Ed\\ y=Cx+f \end{array}$$

where $A=\left[\begin{array}{ll} 0 & 1\\ 0 & a \end{array}\right]$ , $B=\left[\begin{array}{c} 0\\ b \end{array}\right]$ , $C=\left[\begin{array}{ll} 1 & 0\\ 0 & 1 \end{array}\right]$ , $E=\left[\begin{array}{c} 0\\ 1 \end{array}\right]$ , $x=\left[\begin{array}{c} {\tilde{x}}_{i}\\ x_{i} \end{array}\right]$ , $f=\left[\begin{array}{c} {\tilde{f}}_{s}\\ f_{s} \end{array}\right]$

Subsequently, the following result exists.

Theory 5: the perfect decoupling, discussed in Section 3, comes true by the assistant state in (27).

Proof: According to Lemma 1, the proof is straightforward.

Depending on the above discussion, the observer (24) can be used to generate the residual, namely:

(28) $$\{\begin{array}{l} \dot{\hat{x}}=Ax+Bu_{l}+K(y-\hat{y})\\ \hat{y}=C\hat{x} \end{array}$$

So, the residual (25) is obtained, *i.e.*:

(29) $$r(s)=W(y-\hat{y})G_{rd}d+G_{rf}f$$

Theory 6: when $W=[\begin{array}{cc} 1 & 0 \end{array}]$ $K=\left[\begin{array}{ll} \lambda_{1} & 1\\ 0 & a+\lambda_{2} \end{array}\right]$ , the model uncertainty, actuator fault and disturbance in (29) will be decoupled from the residual in (25) perfectly. *λ*_1_ and *λ*_2_ are two positive real numbers.

Proof: According to lemma 1, when $W=\left[\begin{array}{cc} 1 & 0 \end{array}\right]$ $K=\left[\begin{array}{cc} \lambda_{1} & 1\\ 0 & a+\lambda_{2} \end{array}\right]$, it exists *G_rd_* = 0. So:

(30) $$r(s)=W(y-\hat{y})=\frac{s}{s+\lambda_{1}}{\hat{f}}_{s}-\frac{1}{s+\lambda_{1}}f_{s}$$

So, the inverse Laplace transformed residual in (25) is:

(31) $$r(t)=e^{-\lambda_{1}t}{\tilde{f}}_{s}-\frac{1}{\lambda_{1}}(1-e^{-\lambda_{1}t}){\hat{f}}_{s}$$

Then, the fault is detected successfully described by:

(32) $$\underset{t\to \infty }{\lim}r(t)=-\frac{1}{\lambda_{1}}f_{s}$$

From the steady state value of the residual in (32), the information of the target sensor fault is presented intuitively. The size and type of sensor fault could be achieved easily through combining the residual in (32) and the typical mathematical feature of sensor fault.

Remark 10: when the input from the controller couldn't be achieved, the input of the observer is 0. The sensor FD approach provided in this section is still valid. It has no influence on the sensor FD whether the input of the controller in observer (24) exists. The only influence is on the model uncertainty. The feature will make the approach be widely used in the large complex plant.

### 4.3. The Existence of the Assistant State

The integral of state *x_i_* is sure to exist, which is the assistant state *x̃_i_*. When it couldn't be measured by a sensor, the analytical relationship and the measurements from other sensors in the plant could be used to calculate the value of *x̃_i_*. For instance, for each of the vehicle's state, yaw rate, lateral acceleration and steering wheel angle, there are four equations presented by Ding to compute them, respectively [26].

Here, *f̃_s_* denotes the deviation from the actual value, causing by the inexact analytical relationship and other sensors' faults. In order to minimize the negative influence on the residual, a condition was proposed that *f̃_s_* should close to 0 as far as possible. Here, the adaptive threshold method is helpful and easy for usage.

## 5. The Algorithm of Single Sensor's FD and a Simulation Example

### 5.1. The Algorithm of Single Sensor's FD

According to the above results, an algorithm could be proposed for the FD towards one of the sensors in a MIMO non-linear system.


Algorithm 2:Step 1: Determine the target sensor, the proper input and the assistant stateStep 2: Set up the observer which has a same form as (24), design the parameters according to Algorithm 1 when the model uncertainty will disturb the residual easily.Step 3: let
$W=\left[\begin{array}{cc} 1 & 0 \end{array}\right]$
$K=\left[\begin{array}{cc} \lambda_{1} & 1\\ 0 & a+\lambda_{2} \end{array}\right]$and design the parameters *λ*_1_ and *λ*_2_Step 4: stop


### 5.2. Simulation

The above method will be testified in a numerical experiment. Consider a nonlinear system as follows:

$$\{\begin{array}{lll} {\dot{x}}_{1} & = & -x_{2}+x_{4}\\ {\dot{x}}_{2} & = & x_{3}+u_{1}\\ {\dot{x}}_{3} & = & x_{4}^{2}-x_{3}x_{4}-2x_{3}+u_{2}\\ {\dot{x}}_{4} & = & x_{2}-x_{4}\\ y_{1} & = & x_{1}\\ y_{2} & = & x_{2}\\ y_{3} & = & x_{3}\\ y_{4} & = & x_{4} \end{array}$$

where *x*_1_, *x*_2_, *x*_3_ and *x*_4_ are states which are measuring by four sensors(sensor one, sensor two, sensor three and sensor four), *y*_1_*, y*_2_*, y*_3_ and *y*_4_ are outputs, *u*_1_ and *u*_2_ are inputs. Assume that a fault occurs in the sensor measuring the state *x*_4_.

**Figure 2.**
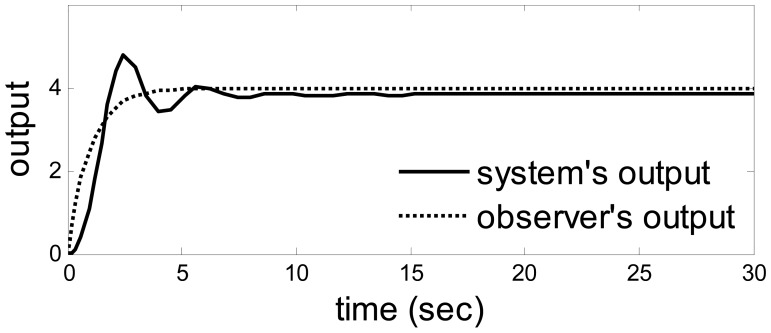
The outputs when *a* = −1 and *b* = 4.

**Figure 3.**
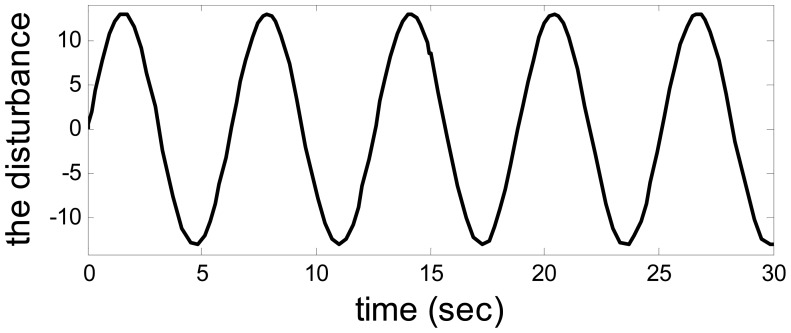
The disturbance.

**Figure 4.**
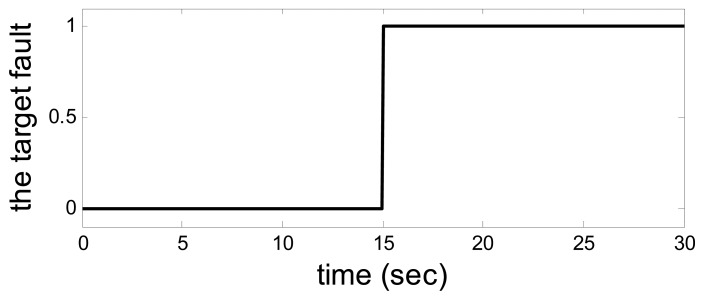
The target fault.

**Figure 5.**
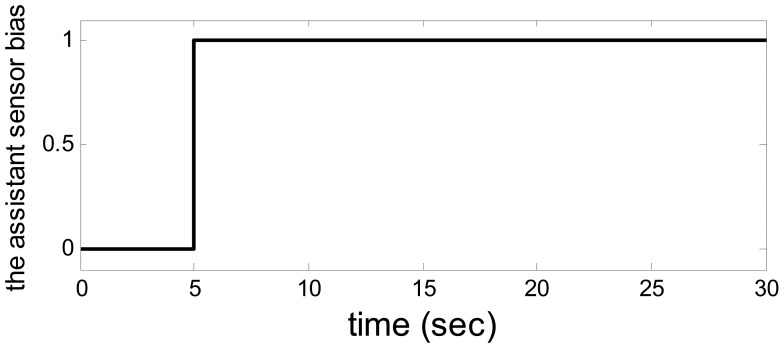
The assistant state bias.

**Figure 6.**
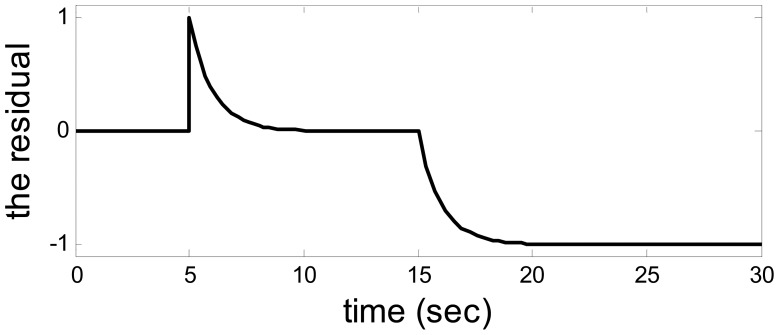
The residual when *a* = −1 and *b* = 4.

Let *a* = −1*, b* = *4,λ*_1_ = *λ*_2_ = 1, *u*_1_ = 1, *u*_2_ = 10. The output from the system and observer are shown in Figure 2. Figure 3 shows the disturbance. The target sensor's fault and the assistant state bias by measuring or estimating are shown in Figure 4 and Figure 5, respectively. As a result, the fault is detected successfully and the residual is shown in Figure 6.

So, when the model uncertainty is not big (Figure 2), the disturbance and model uncertainty are decoupled from the residual perfectly. The assistant state bias is attenuated to 0 and the target fault is detected clearly.

**Figure 7.**
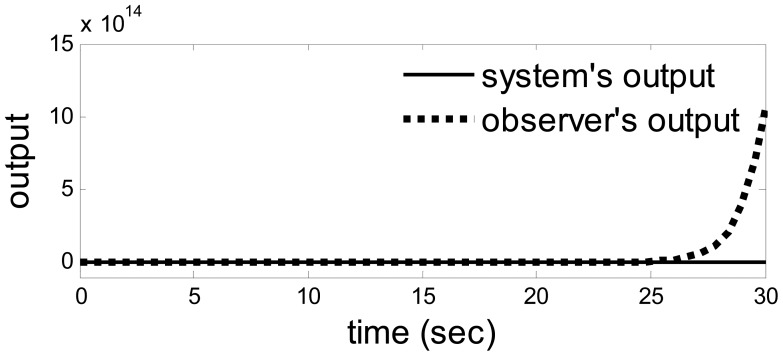
The outputs when *a* = 1 and *b* = 100.

**Figure 8.**
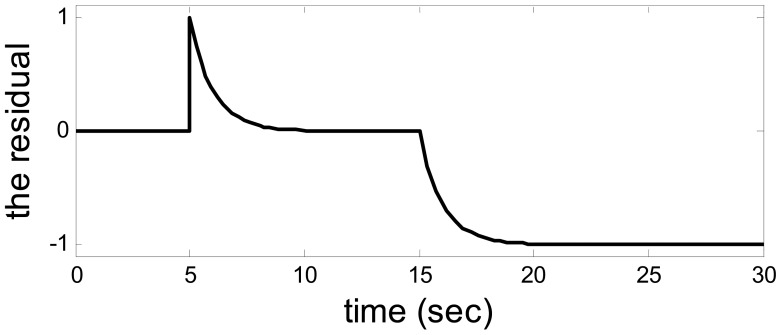
The residual when *a* = 1 and *b* = 100.

When *a* = 1 and *b* = 100, the model uncertainty becomes enormous. This time, the fault could be detected successfully as usual. The outputs from system and observer are shown in Figure 7 in which an enormous model uncertainty emerges. The residual is shown in Figure 8, which is the same as the residual in Figure 6. Hence, when the model uncertainty is very big (Figure 7), the performance of the method is the same as the above. It also means there is a huge space to choose the parameters in the observer.

## 6. The Establishment of the Sensors' FD System and a Simulation Example

Multi-sensor fusion technology was proposed in the 1970s and was applied mainly in military affairs at the beginning. With the fast development of electronic technology, computer technology and sensing technology, multi-sensor fusion technology has been widely used in the fields of robotics, industry control, traffic management and aviation, *etc*. From the engineering aspect, multi-sensor fusion is that in order to finish the decision-making and estimation tasks, based on some principles, integrate and analyze the information from different sources, different modes and different time to get the accurate description of perceived objects [27].

The traditional FD method depends on the information coming from the single source and uses conventional numeral statistical theory. This method will have a heavy workload facing a numerous number of sensors under consideration, and fail to take full advantage of information [28]. Based on the view of multi-sensor fusion technology, it is effective in making full use of the measurement in the sensor network into the sensor FD problem. The process could be divided into two aspects: the design of sensor network and diagnosis logic. According to this view, a sensor fault monitoring system is established in this paper.

### 6.1. The Establishment of the Sensor Network for FD

According to the above discussion, an observer could be set up for the FD towards single sensor of the MIMO plant. Subsequently, a plant's sensors' fault monitoring system could be established for all sensors, by the above algorithm given in every sensor. This time, one sensor in the system maybe not only be diagnosed with the assistant sensors' help, but also plays the role of assistant sensor for others. Synthesizing these, a sensor network could be established for FD.

**Figure 9.**
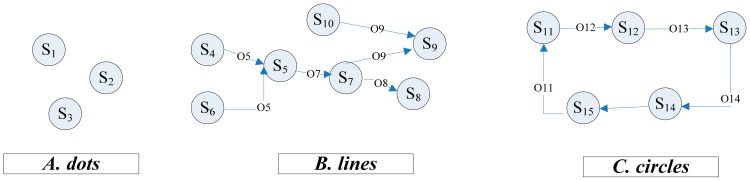
The typical forms of sensor network.

As we know, the assistant state, which could be measured by a sensor or estimated by an analytical relationship. It can be described as:

$${\tilde{x}}_{i}(s_{\text{ass}\_1},s_{ass\_2},\cdots ,s_{\text{ass}\_q})\overset{o_{i}}{\to }x_{i}(s_{i})$$

where *O_i_* denotes the diagnosis observer of state *x_i_*, *s_i_* is the target sensor of *x_i_* , and *s_ass__*_1_*,s_ass__*_2_*,* ⋯ *,s_ass_q_* are all relating sensors for the assistant state *x̃_i_*, *q* = 1,2,3, …. And the order from the assistant sensor to target sensor is called positive order which is also the orientation of the arrow. According to this form, the typical forms of the sensor network could be described in Figure 9.

There are only four styles of sensors maybe exist in the sensor network could be defined as:

**Dot-sensor**: one sensor has no assistant sensors and target sensors
**Head-sensor**: one sensor has no assistant sensors but target sensors
**Foot-sensor**: one sensor has assistant sensors but no target sensors
**Link-sensor**: one sensor has assistant sensors and target sensors

And the network has only *four* styles of structure which are defined as:

**Dots**: only have dot-sensors
**Circles**: only consist of link-sensors and the shape is a circle.
**Lines**: consists of head-sensors, foot-sensors or link-sensors. Along the positive order from one head-sensor to one foot-sensor, the shape is a line.
**Branches**: is one style of the lines attaching to one link-sensor or head-sensor which is in Circles or Lines.

Here, two definitions are given:

**Upstream-sensor**: depending on the positive, one sensor's former sensor is called is upstream-sensor.
**Downstream-sensor**: depending on the positive, one sensor's later sensor is called is downstream-sensor.

### 6.2. The Diagnosis Logic for Sensor Network

Although the residual has strong robust performance, the bias of the assistant state will also exert a negative influence on the residual like disturbance. It means, if *f̃_s_* ≠ 0 and *f_s_* = 0, the residual is not zero immediately. Subsequently, if one of its upstream-sensors has fault, the sensor's residual is not 0 immediately, although its stable state value is 0. As a result, the residual fails to indicate the target sensor's fault in a short time, and the residual in (29) when *f̃_s_* = 0 is:

(33) $$r(t)=\frac{1}{\lambda_{1}}(1-e^{-\lambda_{1}t})f_{s}$$

In order to realize the fault isolation, which means to get a residual which is only influenced by the target sensor's fault, the following basic laws are addressed:

**Law 0:** if all one sensor's (except the head-sensors) assistant sensors' residuals are 0, its residual is only influenced by its fault. With Law 0 and the assumption that only one sensor fault occurs at the same time, the diagnosis logic is presented in the following.
**Law 1:** In Dots—the sensor can't be diagnosed unless it could be brought into the Lines or the Circles by an analytical relationship. The estimation-based method is helpful except for fault tolerance, or even hardwire redundancy.
**Law 2:** In Lines-the residual is not 0 that could be regarded as the indication of the target sensor fault, if all of its upstream-sensors' residual is 0. Head-sensors can't be diagnosed by the observer, but one could use the estimation-based method or hardwire redundancy.
**Law 3:** In Circles-the fault in any one sensor will make all sensors' residuals in the circle not be 0.
  By choosing one sensor as the head-sensor, called quasi-head-sensor, the Circles will transform into Lines and the above Law 2 is effective.
**Law 4:** In branches—depending on that the Link-sensor or Hear-sensor is diagnosed in Circles or Lines, the branch could be treated as a line.

Remark 11: The estimation-based method could indicate the fault effectively and rapidly. The method had used successfully in Electronic Stability Program (ESP) by Ding [18]. Although, the problem of costing, space and workload motive us to develop the analysis redundancy to take the place of the hardwire redundancy, the effectiveness and stability can't meet the practical requirements by only analysis redundancy. It is significant that only use a little hardwires redundancy and lots of analysis redundancy to solve the problem which is dealt with all hardwire redundancy before.

### 6.3. The Establishment of Sensors Fault Monitoring System

**Figure 10.**
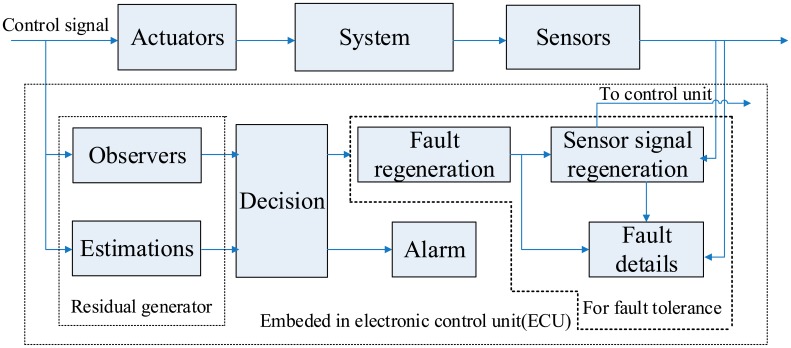
The fault monitoring system.

The fault monitoring system in this paper could make the fault detection, isolation, identification and tolerance to come true. It means, the fault monitoring system could diagnose the fault and handle the fault successfully (Figure 10). According to the above sensor network and the relevant diagnosis logic, the fault monitoring system could be established as follow:

**The observers block**: consists of observers for all Link-sensors and Foot-sensors' FD, sends the residuals to the decision block.
**The estimations block**: consists of estimations for all Dot-sensor, Head-sensors or quasi-head-sensors' FD, and generates residuals by comparing with the measurements. Then send the residuals to the decision block.
**The decision block**: according to the residuals, decides every sensor is fault or fault-free, by pre-set thresholds. The core of the judgment rule is the diagnosis logic (law 1 to law 4). And send the results to the alarm block and fault reconfiguration block.
**The alarm block**: gives the alarm signal to driver, if one fault occurs.
**The fault regeneration block**: utilizes (33) and residual from the observers block, the fault of all Link-sensors and Foot-sensors could be regenerated. A program which gives a pre-process for the residual, like Gaussian noise elimination, could be embedded in this block.
**The sensor signal regeneration block**: utilizes the regenerated sensor signal to take the place of the sensor's wrong signals. And the signals will be sent to the control unit in ECU.

(1) For all Link-sensors and Foot-sensors, the reconfigured signal is:
  $$S_{fault-free}=S_{fault}-f_{s}$$
(2) For all Head-sensors and Dot-sensors, the reconfigured signal is estimated by other sensors' signals and their analytical relationships.
**The fault details block**: collects the information from the faulty sensors, the fault regeneration block and the fault management block. The information could be used by fault tolerance.

### 6.4. Simulation

In order to demonstrate the effectiveness of the fault monitoring system, the same simulation as shown in Section 5.2 is repeated here. The above single sensor FD method wants to prove the effectiveness that the equivalent model instead of the nonlinear system is proper in the view of sensor fault and the problem of perfect decoupling. This section realizes that the whole sensors FD and fault tolerance by building the sensor fault monitoring system.

Considering the nonlinear system in Section 5.2, the following analytical relationship is easy to get:

(34) $$\begin{array}{ccc} \{\begin{array}{ll} x_{4} & =-x_{1}\\ x_{2} & ={\dot{x}}_{4}+x_{4}\\ x_{3} & ={\dot{x}}_{2}-u_{1} \end{array} & \Rightarrow & \{\begin{array}{lll} {\tilde{x}}_{4}(s_{1}) & —\overset{o_{4}}{\to } & x_{4}(s_{4})\\ {\tilde{x}}_{2}(s_{4}) & —\overset{o_{4}}{\to } & x_{2}(s_{2})\\ {\tilde{x}}_{3}(s_{2}) & —\overset{o_{4}}{\to } & x_{3}(s_{3}) \end{array} \end{array}$$

**Figure 11.**
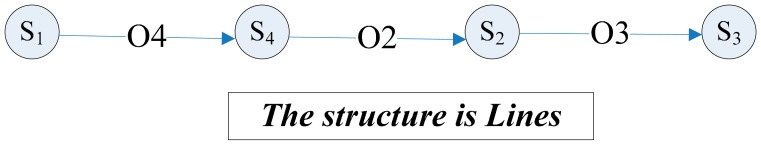
The sensor network structure according to (34).

By establishing the sensor network according to Section 6.1, the Lines structure (Figure 11) could be found and the Law 2 is appropriate.

**Figure 12.**
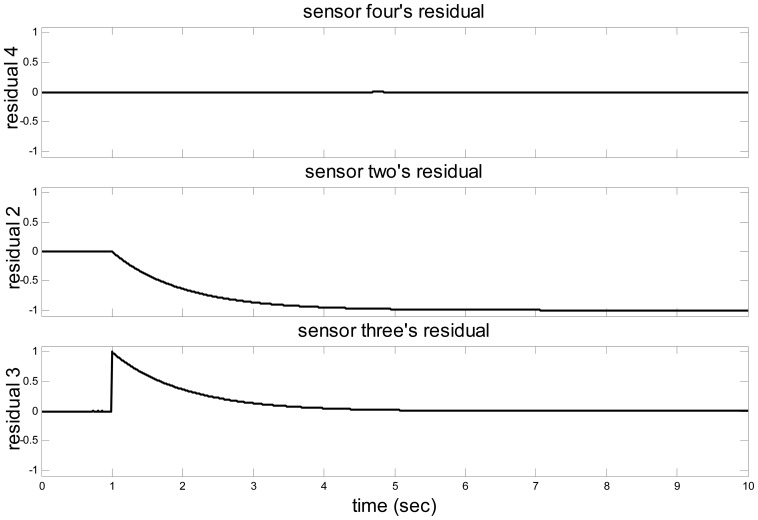
The residuals of sensor four, two and three.

The sensor fault monitoring system could be established. Assuming the sensor one is diagnosed as the Head-sensor fulfilling the Law 2, and a fault occurs in the sensor two. The residual from the observers block shows in Figure 12, and the decision block could give conclusion clearly that a fault has occurred in sensor two. The alarm block will give this information to the driver. Then, the fault regeneration block and the sensor signal regeneration block will start to work rapidly and the regenerated sensor signal will be used to take the place of the faulty sensor's signal. The sensor fault tolerance will be realized and the relating information will be stored in the fault details block.

**Figure 13.**
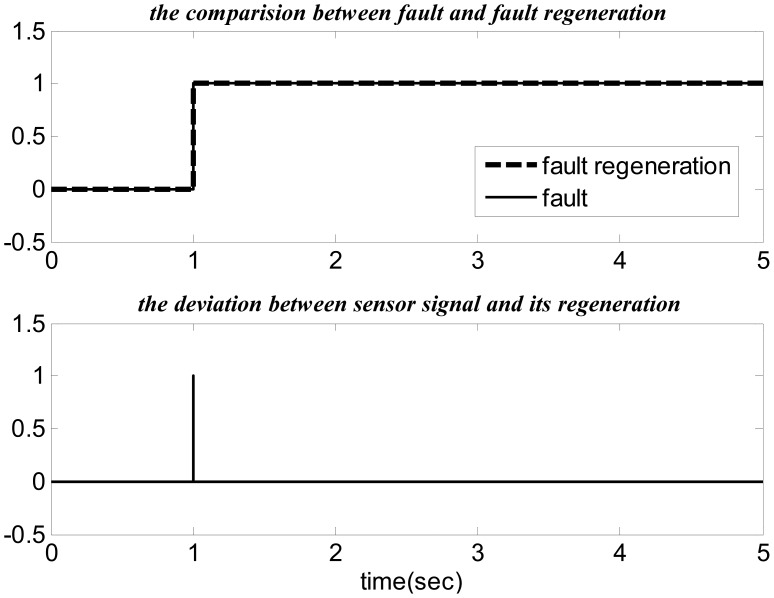
For the fault tolerance.

Figure 13 shows the comparison between fault and fault regeneration and the bias between the sensor signal and the sensor signal regeneration.

## 7. Conclusions

A new model-based sensors FD scheme, using an equivalent model, is developed for a kind of Multiple Inputs Multiple Outputs (MIMO) nonlinear systems which fulfills the Lipschitz condition. A fault monitoring system is established for all sensors' FD and fault tolerance. This method needn't study the nonlinear internal structure character or get the exact model. It gives a new perspective for these problems avoiding complex algorithms and strict additive conditions. It could be widely and easily used in large complex plants with a great deal of sensors, and has profound importance for relating actuators' tolerant control effectively and strongly.
